# On the Treatment of Obesity

**Published:** 1851-03

**Authors:** 


*On the Treatment of Obesity.* (*Corpulence, or Excess of Fat in the Human Body, etc.*) By Dr. T. K. CHAMBERS.

[The subject of corpulence has not met with such consideration as it deserves, we, therefore, have great satisfaction in being able to lay before our readers an extract from a series of papers in which the light of modern science has been most admirably concentrated upon it. Obesity, though too often the subject only of witticism, is in many cases a real evil; and the practitioner will, therefore, gladly avail himself of any rational mode of obviating its inconvenience. In regard to treatment, Dr. Chambers observes:]

That form of disease which commences at birth, and goes on increasing during infancy and childhood, is, I believe, so invariably fatal before the age of puberty, that I do not think we have reason for hoping that it is in any way amenable to medicine. At all events, I have not been able to discover any one whose experience has led him to pronounce it curable. It is a form of monstrosity; and as the subjects of it commonly display some other bodily malformation, and a deficiency of intellect, their death is a relief from a miserable prospect.

When it begins in childhood, or about the time of puberty, we must not be deterred by the circumstance of its being hereditary from attempting to remedy the inconveniences arising from it. We cannot truly reduce our patients entirely to the average size and weight, but we may enable them to pass life in comfort and usefulness.

The later the disease commences, the more controllable it is by management, until the middle period of life is passed, and then old age impedes in some degree the benefit which we may confer, not by rendering our measures inert, but by preventing our employing them quite so actively as we should have done earlier.

The first thing indicated, in all cases, is to cut off, as far as possible, the supply of material. Fat, oil, butter, should be rigorously interdicted in the diet table. But all eatables contain some portion of oleaginous matter, and especially those most convenient to advise the use of for a lengthened period. And almost all are capable of a transformation into fat, when a small quantity of this substance is previously present. It is desirable, therefore, that the mass of food should lie in the stomach as short a time as possible, in order that at least a fatty fermentation may not be set up in it. Very light meals should be taken at times most favorable to rapid digestion, and should consist of substances easy of solution and assimilation. To this end, the time of the meals should be fixed for an early hour in the day, before exertion has rendered the powers of the entrails languid and weak. Breakfast should consist of dry toast, or what is still better, sea-biscuit; and if much active exercise is intended, a small piece of lean meat. Dinner at one, on meat with the fat cut off, stale bread or biscuit, and some plain-boiled maccaroni, or biscuit-pudding, by way of second course.

Liquids should be taken, not at the meal, but half an hour after, so as not to impede the action of the gastric juice upon the mass. Here should end the solid feeding for the day; no second dinner or supper should follow, nor, indeed, any more meals be taken sitting down. A piece of biscuit and a glass of water can be taken standing up, if faintness is experienced; a cup of gruel or roast apple before going to bed.

This is not a scale of diet by any means unattainable. A butcher and retired pugilist has adopted it for some years with the greatest comfort to himself. He is able, upon it, to work in the most violent manner in a small garden which he cultivates for himself in the suburbs. He has reduced himself from 20 to 17 stone; whereas his brother, who has not the same strength of mind, has increased to 23 stone in weight. Persons of more refined education ought, and often do, practise the same self-imposed restraint more easily. J. R.— has reduced himself from 22 to 18 stone, and sometimes brings himself down to 17, but finds that he derives no particular advantage from being of the lower weight.

The smallest amount of nutriment consistent with the health of the individual can be found by experiment only; but we need not fear that ten ounces of solid food a day is too little, for the last-mentioned gentleman confined himself for a long period to that quantity, and found his mental and bodily powers always equal to the strain which the pursuit of a laborious profession in London demands. It may be remarked, by the way, that it is often advisable to add a small allowance of malt liquor at dinner, as otherwise the craving of the appetite is less easily appeased. The beers to be avoided are of course the thick, sweet kinds, but that which is thoroughly fermented, at a low temperature, in the Bavarian way, seems to contain very little injurious matter.

I do not know that any advice concerning sleep is peculiarly applicable to obese persons, beyond what we should recommend to all classes of men. A draught of morning dew, "*nocturni roris auram ante solis ortum bibendam,*" which Aurelian prescribes for the corpulent, is equally beneficial to every one. They are usually uneasy sleepers, and though lethargic, by no means averse to early rising.

In cases where the fat is largely accumulated in the omentum, it is very convenient for the patient to wear a band round the abdomen, which may be tightened gradually. The support thus given to the abdominal muscles relieves the dragging sensation in the loins, which many persons, whose viscera are heavy in proportion to their strength, experience. It enables exercise to be taken with more facility, and appears also by pressure, to afford some assistance to the absorption of fat.

The above remarks will apply equally to all forms of obesity; the abstinence recommended can be borne even by the aged, and only comfort be experienced.

As respects exercise, however, a distinction requires to be made. The young and vigorous, whose obesity does not prevent the use of their legs, cannot employ them more usefully than in walking as long as they are able. The greater number of hours per day that can be devoted to this exercise, the quicker will be the diminution of bulk. But as riding, by the gentle shaking of the abdomen, excites the secretions of the digestive organs more, it should, where practicable, be employed in addition. Where freedom of motion has once been gained, rowing, shooting, any or all of the forms of British gymnastics, should be adopted as regular habits.

But in the asthenic form of the disease, especially in elderly people, this is scarcely practicable. The defect in muscular power prevents the use of the limbs in walking for a long time enough to be advantageous. But where riding can be managed, it should on no account be omitted, and the suspensory belt before mentioned, is often a valuable auxiliary to the employment of this exercise.

The ancients were much more in the habit than we are of using various forms of friction to the skin in treating chronic complaints; and we find in Aurelian a recommendation to the corpulent to employ dry rubbing, either with cloths alone, or with the addition of various powders. Modern habits of cleanliness supersede, in some degree, these remedies. But the skin is not unfrequently greasy from a thick sebaceous secretion, and the circulation through it languid in asthenic obesity, and in these cases horsehair gloves may be used with great advantage. Dr. Flemyng strongly advises friction to be employed to the trunk of the body as promoting absorption and invigorating the surface. The Greek additions of cold bathing or sponging, especially with sea-water, the vapor or hot-air bath, followed by rubbing with salt or with sand, and many other modifications of the same principle enumerated by Aurelian, will naturally suggest themselves to every intelligent patient. The same author very sensibly advises these remedial measures to be employed fasting, and no food to be taken for some time afterwards, and modern habits render before breakfast a convenient time. To these rules of management, medicines, strictly so called, must be viewed as secondary and auxiliary. Unless these laws are obeyed, pharmacopœias are useless.

Purgatives I have generally found not needed in the plethoric form; the bowels usually act once or twice in the day. But in the asthenic obesity *of old people*, where the abdominal walls are weakened by long pressure of an unnatural weight, it is necessary to employ them.

But there is one class of medicines so universally applicable to *all* cases of obesity, that I think a trial of them should never be omitted. The chemical affinity of alkalies for fat, point them out as appropriate alteratives in this complaint, and experience proves that they are suitable to the state of the digestive organs. The most eligible one is liquor potassæ, and it may be administered in much larger quantities than any other. If given in milk-and-water, we may safely commence with half a drachm and raise the dose to a drachm and a drachm and a half three times a day. The milk covers the taste of the potash better than any other vehicle. It has truly the advantage of saponifying a portion of the remedy, but there is no evidence to prove that its efficacy is thereby endangered; indeed soap itself has been strongly recommended. A physician, whose case is recorded by Dr. Flemyng, reduced himself, by alicant soap alone, two stones in weight.

I have often given the above-mentioned doses of liquor potassæ (even to children in cases of scrofula and consumption) without any harm arising from its use, when taken, as desired, in milk. The fear of alkaline medicines has probably arisen from the injury observed by Huxham to follow the use of Mrs. Stephen's saponaceous mixture, at one time so popular, and therefore often misapplied. The injury appears to have originated from their having been employed in improper cases, such as debilitated gouty subjects, chronic stone in the bladder, and the like, to which, of course, much harm would be done.

A poor woman who sold eggs in Chelsea, was becoming quite unable to gain her livelihood by her ordinary occupation. I have not kept a note of her weight and height, and therefore she is not mentioned in the table of cases, but she was extremely obese, and the cause of a variety of symptoms she complained of seemed traceable entirely to the accumulation of fat. By taking liquor potassæ only, without change of diet, she was reduced so far as to carry on her trade with comfort.

Another case was communicated to me the other day, of a gentleman who weighed 19 stone and 7 lbs. By regimen, exercise, and liquor potassæ, he was reduced two stone and a half in six weeks.

I have mentioned bleeding, and perhaps that may cause some surprise, after the observations which have been made on the state of the circulation in fat people. But where distinct signs of plethora are present—such as pain over the eyebrows, beating of the temples, restless sleep by night, lethargy by day, with full lips and an elastic skin—it is capable of being employed with safety; and where it is employed, the advantage derived at the commencement of a course of treatment is very great, for it gives all the other remedies a fair start; and by affording immediate relief to many symptoms, gives the patient a favorable opinion of the plan he has undertaken.

On the other hand, it is scarcely necessary to say, that much risk attends the loss of blood; for if the heart has become atrophied and weak, it will not stand the shock. Venesection may cause either sudden death, from failure of the heart's action, or effusion of blood in the brain, from disturbance to the circulation.

Bitter tonics are often of great advantage in enabling the stomach to digest more easily and rapidly, and therefore to be contented with a smaller quantity of really nourishing food. The increase of appetite which they cause does no harm; for when patients are getting better, they are usually more obedient to their medical man, and can be taught to control it. Gratitude for the benefit they have received, makes them glad to follow advice, however hard.

Some medicines must now be mentioned, which have been recommended for the cure of obesity, but which analogy and experience do not approve.

Vinegar has been employed by those who are foolish enough to practise upon themselves; but as it produces thinness only by injuring the digestive organs, the benefit is not worth the price paid for it, and no medical man would ever advise the use of such a remedy.

Iodine has been spoken of as likely to do good, from the power it exhibits of stimulating the absorbents in cases of scrofula and tumors. But its moderate use certainly does not cause the disappearance of healthy fat. Indeed it has been noticed by Lugol, and is matter of daily observation at our metropolitan hospitals, that patients frequently acquire a considerable degree of embonpoint during the time they are taking iodine. The cases of tumors and of fats are very distinct. As Dr. Pereira remarks, "the enlargements which these agents (mercury and iodine) remove, are not mere hypertrophies; their structure is morbid, and they must in consequence have been induced by a change in the quality of the vital activity; in other words, by morbid action. Medicines, therefore, which remove these abnormal conditions, can only do so by restoring healthy action." But the action which causes the deposition of fat in the adipose tissue is, though excessive, of a healthy nature, and harm, rather than benefit, is to be expected from the medicine under discussion; that harm which always accrues from a valuable remedy wrongly employed.—*Half Yearly Abstract of the Medical Sciences.*

